# Neuropharmacological, Antidiarrheal, and Antimicrobial Effects of *Chaetomorpha aerea* Acetone Extract: GC-MS Profiling and In Silico Analysis

**DOI:** 10.1155/sci5/6745529

**Published:** 2025-07-24

**Authors:** Md. Mahmudul Hasan, Md. Safayat Hossen Momen, Md. Abdul Alim, Sajjad Hossen Chowdhury, Miton Chowdhury, Md Al Mamun, Fatema Tuz Zohra, Md. Jakaria Parvez, Suman Das, S. M. Moazzem Hossen

**Affiliations:** ^1^Department of Pharmacy, Faculty of Biological Sciences, University of Chittagong, Chittagong 4331, Bangladesh; ^2^Department of Pharmacy, University of Science and Technology Chittagong, Chattogram 4202, Bangladesh; ^3^Department of Pharmacy, University of Asia Pacific, Dhaka, Bangladesh; ^4^Department of Pharmacy, North South University, Dhaka, Bangladesh; ^5^Department of Pharmacy, University of Development Alternative, Dhaka, Bangladesh; ^6^Chattogram Laboratories, Bangladesh Council of Scientific and Industrial Research (BCSIR), Chattogram 4220, Bangladesh

**Keywords:** antidiarrheal agent, antimicrobial, *Chaetomorpha aerea*, edible seaweed, GC-MS analysis, marine algae, neuromodulator, phytochemicals

## Abstract

*Chaetomorpha aerea*, distributed in temperate and tropical coastal regions, is traditionally consumed as a nutrient-rich food source in coastal communities and is believed to possess medicinal properties. This study evaluated the sedative, anxiolytic, antidiarrheal, and antimicrobial activities of the acetone extract of *C. aerea* (AECA). Furthermore, GC-MS performed a quantitative phytochemical analysis of the AECA. Sedative activity was evaluated in mice using the open field test (OFT) and hole cross test (HCT); anxiolytic activity was assessed using the elevated plus maze (EPM), hole board test (HBT), and light–dark box test (LDBT); and antidiarrheal activity was determined through castor oil–induced diarrhea and gastrointestinal motility test. The disc-diffusion method was employed to evaluate antibacterial activity. In both OFT and HCT models, AECA 400 mg/kg demonstrated a significant reduction of square crossed and hole crossed compared to diazepam, respectively. In the case of EPM and HBT, 400 mg/kg dose of AECA demonstrated significant dose-dependent activity. Both in the castor oil–induced diarrhea and gastrointestinal motility test, 400 mg/kg of AECA demonstrated moderate inhibition of diarrhea compared to standard loperamide. Antimicrobial assay of AECA showed considerable inhibition against *Escherichia coli* and *Salmonella typhi*, measuring inhibition zones of 14 mm and 13 mm. Bioactive metabolites from GC-MS analysis were investigated through molecular docking. Docking was performed against GABA_A_ receptor (6X3T), MAO-A (2Z5X), M3 muscarinic receptor (5ZHP), *E. coli* FabI (1LX6), and GyrA (5ZTJ). AECA showed notable sedative, anxiolytic, antidiarrheal, and antimicrobial activities. To evaluate the molecular pathways involved and isolate the bioactive ingredients, more research is required.

## 1. Introduction

Seaweed, also known as marine macroalgae, has long been an integral part of the human diet and traditional medicine because of its nutritional, phytochemical, and medicinal values [1]. They are recognized medicinally for a variety of bioactive substances, including vitamins, sulfated polysaccharides, flavonoids, and polyphenols [2]. Antioxidant, anti-inflammatory, antimicrobial, and anticancer properties of seaweeds are exhibited in many studies [3]. Notably, they also exhibit anxiolytic and sedative actions by modulation of the neurotransmitter system [4]. By enhancing gut health and inhibiting the pathogenic bacteria due to the high percentage of polysaccharides, they alleviate diarrhea [5].

Anxiety and insomnia are prevalent health issues affecting more than 40 million adults in the United States every year. Anxiety is characterized by excessive worry, fear, and psychological arousal in individuals and is denoted as the most prevalent mental illness in the United States [6]. Insomnia is marked by difficulties in falling and staying asleep, impacting around 30% of adults, causing significant impairment in daily performance, cognitive performance, and overall quality of life [7]. On the other hand, diarrhea, characterized by loose, watery stool and abdominal discomfort, is a frequent gastrointestinal issue that is the primary cause of mortality and morbidity globally, particularly in developing countries. It accounts for a significant childhood death due to malnutrition and dehydration [8]. The prevalence of antibiotic resistance makes bacterial illnesses a major issue since they are harder to treat. The development of genetic resistance in bacteria renders standard antibiotics ineffective, increasing morbidity, lengthening hospital stays, and driving up healthcare costs [9]. Antibiotic overuse and misuse worsen this problem by limiting the available treatments for illnesses like sepsis, pneumonia, and tuberculosis and endangering public health worldwide [10].

Current therapies for these disorders frequently include pharmaceutical interventions. Benzodiazepines and selective serotonin reuptake inhibitors (SSRIs) are common treatments for anxiety and insomnia, although they can cause drug dependency, dizziness, weight gain, and withdrawal symptoms [11]. Antibiotics and antidiarrheal drugs are commonly used to treat diarrhea; however, they can have side effects such as antibiotic resistance, constipation, and disruption of natural gut flora [12]. Because of these negative effects, there is an increased interest in natural product–based treatments, as they have fewer adverse effects [13].

*Chaetomorpha aerea*, a green algae species, is naturally distributed in the temperate and tropical coastal regions, which are morphologically unbranched, cylindrical filaments with elongated cells varying in color from bright to dark green [14]. It has nutraceutical and medicinal values because of its diversified composition of various components. *C. aerea* is an important dietary ingredient since it is high in proteins, vitamins, and minerals, including iodine, iron, calcium, and vitamins A and C. It is utilized as a dietary component in coastal communities across temperate and tropical regions, particularly in South and Southeast Asia [15]. Because of its polyphenols, flavonoids, and sulfated polysaccharides, it has been found to have notable antibacterial, antioxidant, and anti-inflammatory potentials in medicine. Interestingly, it inhibits harmful bacteria and improves gut health, having antidiarrheal effects. Moreover, traditional applications for calming nerves, reducing anxiety, promoting sleep, treating restlessness, and soothing stress-associated digestive complaints have also been pronounced [16]. Additionally, bioactive chemicals that interact with the central nervous system to reduce anxiety and promote relaxation are the source of its anxiolytic and sedative activities [4]. These characteristics underline its medicinal potential and call for additional pharmacological research.

Nonetheless, despite its traditional application and documented bioactivities, direct in vivo experimental evidence substantiating the effectiveness of *C. aerea* extracts against anxiety, insomnia, diarrhea, and bacterial infections is currently lacking. This study aims to investigate sedative, anxiolytic, antidiarrheal, and antimicrobial potentials of the acetone extract of *C. aerea* (AECA), both in vivo and in silico approaches.

## 2. Materials and Methods

### 2.1. Collection and Preparation of Extract

The algae *C. area* was collected from Kutubdia Island, Bangladesh, and authenticated by Mr. Mohammad Forkanul Hamid, Department of Fisheries, University of Chittagong (Accession No. CU/DP/2023/03). After collection, the sample was rinsed with fresh water to remove residual sand and salt, then air-dried for 10 days. The dried sample was ground into coarse particles using a mechanical grinder, soaked in acetone for 14 days with intermittent shaking, and filtered through Whatman filter paper No. 1. The filtrate was concentrated using a Büchi rotary evaporator under reduced pressure, maintaining the temperature below 50°C. A crude extract yield of 12 g (10% extraction efficiency) was obtained and stored at 4°C for further use.

### 2.2. Quantitative Phytochemical Analysis

#### 2.2.1. Total Phenol Content (TPC) and Total Flavonoid Content (TFC)

Mosoh et al. used the Folin–Ciocalteu reagent to determine the TPC of AECA, measuring absorbance at 765 nm with gallic acid as the standard. The TFC was assessed using AlCl_3_ and CH_3_CO_2_K, with absorbance at 415 nm, referencing quercetin equivalents [17].

#### 2.2.2. Gas Chromatography–Mass Spectrometry (GC-MS) Analysis

The AECA was analyzed using a Shimadzu TQ 8040 mass spectrometer with EI ionization, coupled with a Shimadzu GC-17A gas chromatograph and an Rxi-5 ms capillary column. Helium was used as the carrier gas at 0.6 mL/min, with a GC-MS interface temperature of 280°C, a scan range of 40–350 amu, and compound identification via the NIST GC-MS library (version 08-S).

### 2.3. Drugs and Chemicals

The chemicals and solvents utilized in this experiment were all of analytical grade. Diazepam and loperamide were purchased from Square Pharmaceutical Limited, a renowned Bangladeshi pharmaceutical company. Other analytical grade chemicals and reagents were provided by the Department of Pharmacy, University of Chittagong.

### 2.4. Experimental Animal

Albino mice (4-5 weeks old, 20–25 g) were obtained from BCSIR, Chittagong, and acclimatized for one week before testing. The animals were maintained in the animal facility of the Department of Pharmacy at the University of Chittagong, Bangladesh, under regulated conditions. These conditions included a 12-h light–dark cycle at 25 °C ± 2°C and a relative humidity of 45%–55%. The enclosures were sterile and dry. A standard diet and pure water were provided, with food deprivation occurring for 12 h before and during the experiment.

### 2.5. Animal Euthanasia

The study was approved by the Ethical Review Board (ERC/DPCU/2024/03), adhering to Swiss Academy of Sciences guidelines and the 2013 Animal Euthanasia Guidelines. Also, in vivo experiments comply with the ARRIVE guideline checklists.

### 2.6. Acute Oral Toxicity Test

An acute toxicity investigation was conducted to ascertain the LD_50_ of the extracts, by OECD criteria [18]. Each toxicity test comprised five animals that were subjected to overnight fasting before the administration of the extract. The animals were administered a single oral dose of AECA at 500, 1000, 1500, or 2000 mg/kg body weight. Food was withheld for an additional 3–4 h following treatment. Observations were undertaken rigorously for the initial 30 min, followed by vigilant monitoring over 24 h (particularly during the first 4 h), then intermittently for 3 days to evaluate delayed toxicity. Daily assessments documented adverse effects, encompassing alterations in integument, pelage, ocular condition, mucosal membranes, circulatory function, respiratory activity, autonomic responses, and central nervous system function [19]. The therapeutic dose was established as one-tenth of the median lethal dose with Karber's arithmetical technique, alongside the Hodge and Sterner scale (LD_50_ > 2.0 g/kg) [20].

The LD_50_ was determined using the equation

(1) $$\begin{array}{c} {\text{LD}}_{50}=\frac{{\text{LD}}_{100}-\sum \left(a\times b\right)}{n}, \end{array}$$

here, *n* = the total count of animals in a group, *a* = the dosage variation between two successive administrations of the extract/substance, *b* = the mean number of fatalities recorded after two consecutive doses, and LD_100_ = the lethal dose that results in total mortality among the test subjects.

### 2.7. Experiment Design

Swiss albino mice were divided into control, standard, and test groups, receiving 1% Tween 80, AECA (200 and 400 mg/kg, oral) based on the acute oral toxicity test described in Section 2.6, diazepam (1 mg/kg, i.p.), or loperamide (5 mg/kg, i.p.) for various behavioral and gastrointestinal tests. From acute toxicity study, toxicity was observed at the dose of 2000 mg/kg of AECA. Following Karber's arithmetical technique, one-tenth of the toxic dose, 200 mg/kg, was selected for the safest dose. To observe the dose–response relationship, double the safest dose, 400 mg/kg, was also administered.

### 2.8. Sedative Activity

#### 2.8.1. Open Field Test (OFT)

The sedative-anxiolytic effect of AECA was determined using behavioral metrics such as the number of square movements, as described by Saleem et al. [21]. The open field apparatus was a square box (60 × 60 × 60 cm) with 25 equal squares (5 × 5 cm) marked in black and white. The mice received the treatments indicated in Section 2.6. Following administration, each mouse was separately placed in the device at intervals of 0, 30, 60, 90, and 120 min and watched for 3 min.

#### 2.8.2. Hole Cross Test (HCT)

The HCT was conducted using a device (30 × 20 × 14 cm) with a partition and a hole (3 cm in diameter) positioned 7 cm above the floor, using a modified method published by Subhan et al. [22]. Mice were treated in the manner described in Section 2.6. Following treatment, each mouse was placed individually in the device, and the total number of crosses through the hole was counted over 3 min at 0, 30, 60, 90, and 120 min.

### 2.9. Anxiolytic Activity

#### 2.9.1. Elevated Plus Maze (EPM)

The EPM test was used to assess antianxiety behavior. The apparatus was composed of two open arms (35 × 5 cm^2^), two closed arms (35 × 20 cm^2^), and a central square (5 × 5 cm^2^) elevated 25 cm above the ground. The mice received the treatments indicated in Section 2.6. Each mouse was placed in the EPM's central square 60 min after being administered. The observations were recorded for 5 min. The percentage of time spent and entry into the open arms were computed using the following formulas [23]:

(2) $$\begin{array}{c} \%\text{ of time spent in open arm}=\frac{\text{Time spent in open arms}}{\text{Time spent in open arm}+\text{Time spent in closed arm}}\times 100,\\ \%\text{ of entry in open arm}=\frac{\text{Entry in open arms}}{\text{Entry in open arm}+\text{entry in closed arm}}\times 100. \end{array}$$

#### 2.9.2. Hole Board Test (HBT)

The HBT was employed to assess anxiolytic activity, based on head dipping behavior as a measure of anxiety and exploratory activity [24]. The device was a wooden board (20 × 40 cm) with 16 holes spaced evenly apart. Treatments were given to mice under Section 2.6. Each mouse was kept in the center of the device 30 minutes after injection. Over 5 minutes, the number of head dips as well as the initial head dip's delay were noted.

### 2.10. Antidiarrheal Activity

#### 2.10.1. Castor Oil–Induced Diarrhea

The experiment began with an 18-hour fast for the mice. The administration of treatments followed Section 2.10 guidelines. Each rodent was given 0.5 mL of castor oil orally through gavage 1 hour after treatment. Each mouse was housed in a cage along with blotting paper lining it, and for 4 hours, the quantity of both wet and dry excrement was counted every hour. At the beginning of every hour, fresh blotting paper was provided. The following formula was used to determine the % inhibition of antidiarrheal activity [25]:

(3) $$\begin{array}{c} \%\text{ Inhibition}=\left[\frac{\left(A-B\right)}{A}\right]\times 100, \end{array}$$

where *A* = average number of diarrheal feces in the control group and *B* = average number of diarrheal feces in the treated group.

#### 2.10.2. Charcoal Marker Gastrointestinal Motility Test

The mice were dealt with per Section 2.6. Each mouse was given 1 mL of a charcoal solution (10% charcoal, 5% gum acacia) orally via gavage an hour after treatment. An hour later, the mice were euthanized by administering pentobarbital (200 mg/kg) via intraperitoneal (IP) injection. Measurements were made of the small intestine's overall length and the charcoal marker's trip distance. The following formula was used to determine the % inhibition of antidiarrheal activity [26]:

(4) $$\begin{array}{c} \%\text{ Inhibition}=\left[\frac{\left(A-B\right)}{A}\right]\times 100, \end{array}$$

where *A* = distance traveled by the charcoal in the control group (cm) and *B* = distance traveled by the charcoal in the test group (cm).

(5) $$\begin{array}{c} \text{Peristalsis index}=\left(\frac{\text{Distance traveled by the charcoal}}{\text{Total length of the small intestine}}\right)\times 100. \end{array}$$

### 2.11. Antibacterial Assay

Utilizing the disc-diffusion method, the antibacterial screening was conducted as described in Lorian [27]. Standard pefloxacin (5 μg/disc) and sample discs containing AECA 25 μg/disc each were used as positive controls, while blank discs were used as the negative control. The higher dosage of *C. aerea* extract (25 μg) relative to pure pefloxacin (5 μg) can be attributed to the composition of crude extracts consists of diluted active chemicals within a complex matrix, requiring higher concentrations to attain bioactivity compared to purified antibiotics and standard antimicrobial testing for natural products typically employs elevated dosages (20–100 μg/disc) to facilitate dilution, adhering to established phytochemical screening methodologies [28, 29]. The cultured organisms were *Escherichia coli, Salmonella typhi, Pseudomonas aeruginosa,* and *Staphylococcus aureus*.

### 2.12. In Silico Studies

#### 2.12.1. Ligand Preparation

The GC-MS analysis of the acetone extract of the seaweed *C. aerea* revealed a total of 12 small metabolites. These molecules were obtained in 3D SDF format from the PubChem database for docking purposes. If the 3D SDF format was not available, Open Babel software was used to download and convert the 2D SDF file to 3D SDF [30]. Before docking simulation, all ligands were minimized and saved in .pdbqt format using AutoDock Tools (version 1.5.6) [31].

#### 2.12.2. Protein Preparation

For sedative, anxiolytic, antidiarrheal studies, human GABA_A_ receptor alpha1-beta2-gamma2 subtype (PDB ID: 6X3T), human monoamine oxidase A (PDB ID: 2Z5X), and M3 muscarinic acetylcholine receptor (PDB ID: 5ZHP), and for antibacterial activity, *E. coli* enoyl reductase NAD+ (PDB ID: 1LX6) and gyrase A (PDB ID: 5ZTJ) were sourced from the RCSB Protein Data Bank (https://www.rcsb.org/structure) in PDB format. Water molecules and other heteroatoms were erased from the protein structures using Discovery Studio 2020 (Studio, 2008). After that, the proteins underwent energy minimization using the conjugate gradient and steepest descent techniques in Swiss Viewer for PDB (Version 4.1.0) [32]. The PDB files were converted to .pdbqt format with AutoDock Tools (version 1.5.6) as well, and finally stored in this format.

#### 2.12.3. Molecular Docking Analysis

The docking of the selected proteins with *C. aerea's* ligands was executed using PyRx AutoDock Vina. A semiflexible docking system was employed for this analysis, where the protein was rigid and the ligands were flexible. AutoDock specified the parameters that defined the box type and formed the grid box. A well-known inhibitor, the co-crystallized ligand, attaches itself to important residues in the protein's active site. The entire binding pocket was encircled by a grid box. The procedure was verified by redocking the reference ligand into the protein's binding site before docking fresh ligands. The crystallographic pose that was established experimentally was then aligned with the lowest-energy pose produced by the docking program. The two poses' root mean square deviation (RMSD) was computed, with less than 2 Å serving as an acceptable cutoff [33]. After the validation of docking procedure, the grid box of dimensions (25 × 25 × 25) Å, including the grid box center, was set to ‘X = 133.12,' ‘Y = 117.05,' and ‘Z = 154.00' (RMSD = 1.05 Å) for the human GABA_A_ receptor alpha1-beta2-gamma2 subtype (PDB ID: 6X3T), ‘X = 40.66,' ‘Y = 24.66,' and ‘Z = − 14.74' (RMSD = 0.75 Å) for human monoamine oxidase A (PDB ID: 2Z5X), ‘X = − 23.73,' ‘Y = − 48.14,' and ‘Z = 195.04' (RMSD = 0.37 Å) for M3 muscarinic acetylcholine receptor (PDB ID: 5ZHP), ‘X = − 5.93,' ‘Y = 42.58,' and ‘Z = 158.17' (RMSD = 0.82 Å) for *E. coli* enoyl reductase NAD+ (PDB ID: 1lx6), and ‘X = 38.22,' ‘Y = 29.18,' and ‘Z = −5.83' (RMSD = 1.35 Å) for gyrase A (PDB ID: 5ztj). Moreover, BIOVIA Discovery Studio Visualizer 2020 (Biovia, 2017) was employed to construct two- and three-dimensional docking interactions.

#### 2.12.4. ADME Evaluation

The ADME properties of key AECA bioactive metabolites were evaluated using Lipinski's Rule of Five via SwissADME and pkCSM web server.

### 2.13. Statistical Analysis

Data are represented as mean ± SEM (standard error of the mean). Statistical significance compared to the control group was determined using Dunnett's *t*-test (^∗^*p* < 0.05, ^∗∗^*p* < 0.01, and ^∗∗∗^*p* < 0.001). SPSS (version 25) was used to analyze data, and GraphPad Prism version 8.0 was employed for graph generation. The in vivo experiments employed 5 mice in every group.

## 3. Result

### 3.1. Quantitative Phytochemical Constituents

#### 3.1.1. Total Phenolic and Flavonoid Content

The total phenolic content (20.93 ± 1.05 mg GAE/g extract) was higher than that of flavonoids (15.78 ± 0.70 mg QUE/g extract) shown in Table 1.

#### 3.1.2. GC-MS Analysis of *C. aerea*

**Figure 1:**
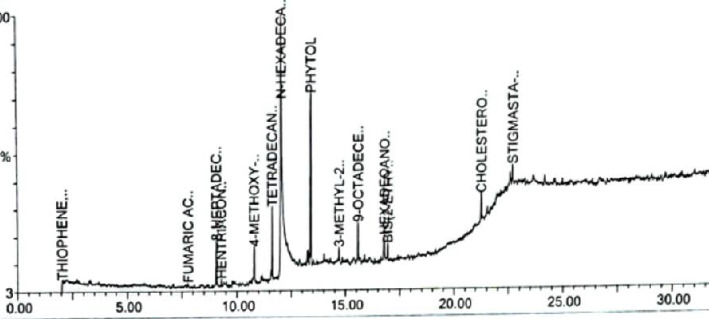
GC-MS chromatogram of acetone extract of *C. aerea* (AECA). A total of 12 metabolites were eluted with retention times ranging from 2.5 to 32 min from the AECA sample.

A total of 12 metabolites were eluted between 2.5 min and 32 min retention time from the AECA sample (Figure 1). Based on the NIST GC-MS library version 08-S, these metabolites were identified (Table 2).

### 3.2. Sedative Activity

#### 3.2.1. OFT

**Figure 2:**
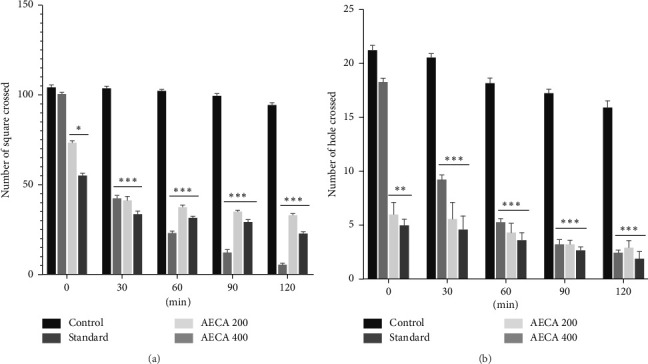
Sedative activity of *C. aerea* on the open field test (a) and hole cross test (b). In the open field and hole cross tests, the number of squares and holes crossed were measured at the point of 0, 30, 60, 90, and 120 min after treatment, respectively. Mean ± SEM (*n* = 5), with ^∗^*p* < 0.05, ^∗∗^*p* < 0.01, and ^∗∗∗^*p* < 0.001 indicating statistical significance.

AECA (200 and 400 mg/kg) exhibited a dose-dependent locomotor activity which was statistically significant (*p* < 0.001) compared to control (1% Tween 80) (Figure 2).

#### 3.2.2. HCT

The AECA (200 and 400 mg/kg) showed a significant (*p* < 0.001) dose-dependent hole-crossing ability of mice compared to the control (Figure 2).

### 3.3. Anxiolytic Activity

#### 3.3.1. EPM

**Figure 3:**
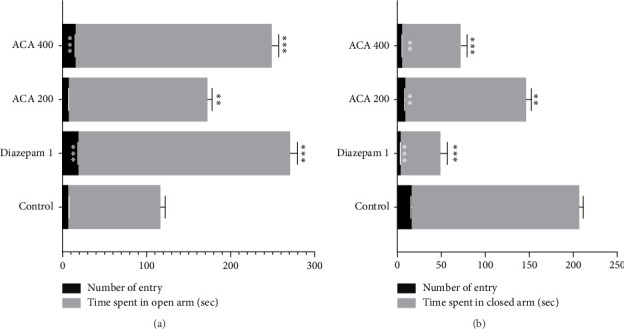
Anxiolytic activity of AECA in elevated plus maze (open arm (a) and closed arm (b)). The number of entries and time spent in both arms are depicted in the illustration. Mean ± SEM (*n* = 5), with ^∗∗^*p* < 0.01, and ^∗∗∗^*p* < 0.001 indicating statistical significance.

The AECA 200 mg/kg and 400 mg/kg significantly increased the time spent and number of entries in the open arm compared to the control group in a dose-dependent way (Figure 3).

#### 3.3.2. HBT

**Figure 4:**
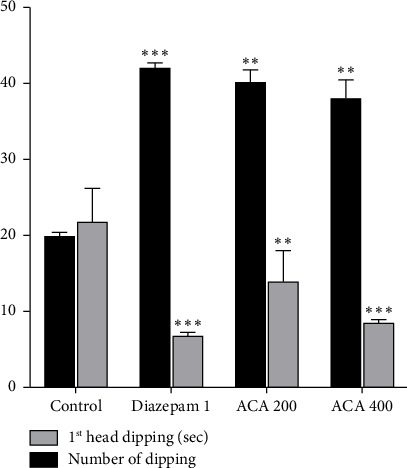
Anxiolytic activity of AECA in hole board test. Time required for the first head dip and the number of head dips are illustrated in the figure. Mean ± SEM (*n* = 5), with ^∗∗^*p* < 0.01, and ^∗∗∗^*p* < 0.001 indicating statistical significance.

The number of head dips increased in a dose-dependent manner in the AECA 200 mg/kg and 400 mg/kg significantly compared to the control (Figure 4).

#### 3.3.3. Light–Dark Box Test (LDBT)

**Figure 5:**
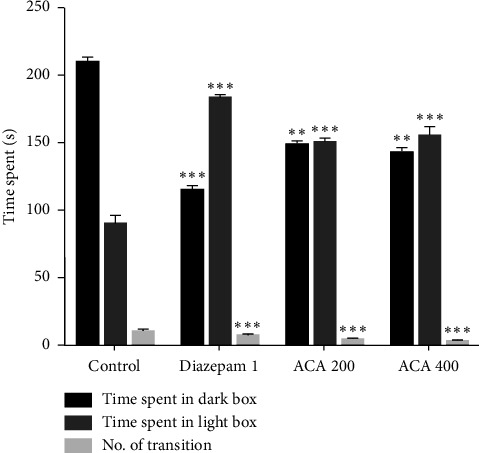
Anxiolytic activity of AECA in the light–dark box test. Time spent in the light and dark boxes, along with the number of transitions between them, are depicted in this figure. Mean ± SEM (*n* = 5), with ^∗∗^*p* < 0.01, and ^∗∗∗^*p* < 0.001 indicating statistical significance.

In this experiment, there were a significant number of transitions to the light compartment. The data is given in Figure 5.

### 3.4. Antidiarrheal Test

#### 3.4.1. Castor Oil–Induced Diarrhea

The AECA significantly inhibited dose-dependent diarrhea and defecation, which is summarized in Table 3.

#### 3.4.2. Gastrointestinal Motility

AECA 400 mg/kg significantly decreased the peristaltic index by 54.55 ± 0.53% (*p* < 0.01), whereas the standard loperamide 5 mg was 42.66 ± 0.36 (*p* < 0.001) (Table 4).

### 3.5. Antimicrobial Activity

**Figure 6:**
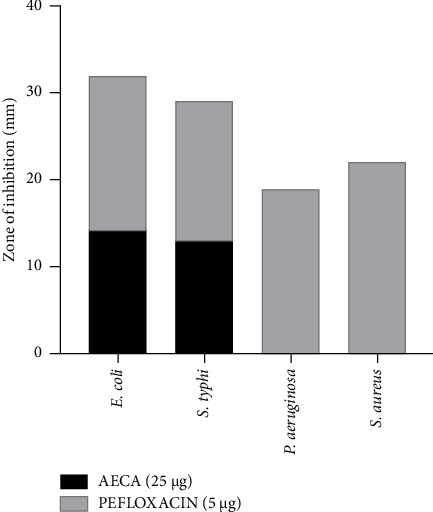
Zone of inhibition against different bacterial species. AECA showed moderate inhibition against *E. coli* and *S. typhi* but weaker activity while pefloxacin exhibited strong, broad-spectrum efficacy.

The antimicrobial potential of the AECA (25 μg) was analyzed in this study, where it demonstrated moderate antimicrobial activity. Out of four bacteria, it exhibited its activity against *E. coli* and *S. typhi.* The zone of inhibition against two Gram-negative bacteria, *E. coli* and *S. typhi*, was 14 mm and 13 mm, while standard pefloxacin (5 μg) inhibited 18 mm and 16 mm, respectively (Figure 6).

### 3.6. In Silico Study

#### 3.6.1. Molecular Docking Study

**Figure 7:**
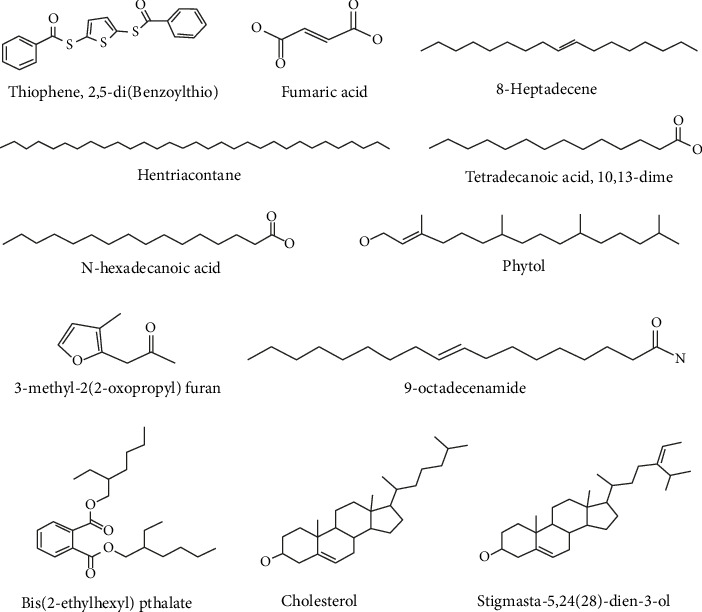
2D structures of the GC-MS compounds.

Metabolites found in the AECA GC-MS study were docked against five major proteins: human monoamine oxidase A (PDB ID: 2Z5X), human GABAA receptor alpha1-beta2-gamma2 subtype (PDB ID: 6X3T), and M3 muscarinic acetylcholine receptor (PDB ID: 5ZHP) for anxiolytic, sedative, and antidiarrheal activities, and for antibacterial activity, *E. coli* enoyl reductase NAD+ (PDB ID: 1LX6) and gyrase A (PDB ID: 5ZTJ). The binding energy (kcal/mol) for every protein is depicted in Table 5. The 2D structure of the GC-MS compound is illustrated in Figure 7.

##### 3.6.1.1. Molecular Docking Related to Sedative Activity

**Figure 8:**
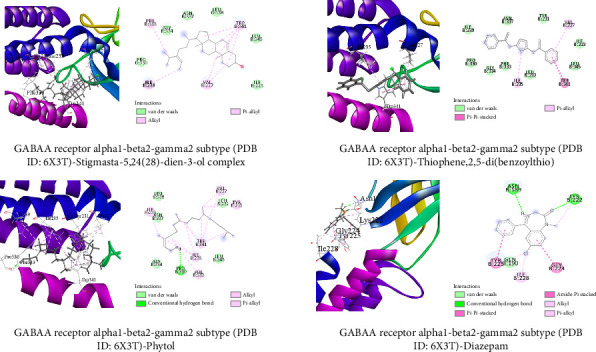
Molecular docking interactions of the top three metabolites and standard (diazepam) with human GABAA receptor alpha1-beta2-gamma2 subtype (PDB ID: 6X3T). Top three metabolites and standard drugs bind with the amino acids at the active site of the protein with varying types of bonds indicated in the figures.

The top three docked compounds are illustrated (both 3D and 2D) in Figure 8. Stigmasta-5,24(28)-dien-3-ol was the compound that showed the highest binding score of −7 kcal/mol with human GABAA receptor alpha1-beta2-gamma2 subtype (PDB ID: 6X3T), while the standard diazepam depicted a binding score of −5.7 kcal/mol. The other two top metabolites, thiophene,2,5-di(benzoylthio) and phytol, bound with an energy of −6.3 and −5 kcal/mol, respectively.

##### 3.6.1.2. Molecular Docking Related to Anxiolytic Activities

**Figure 9:**
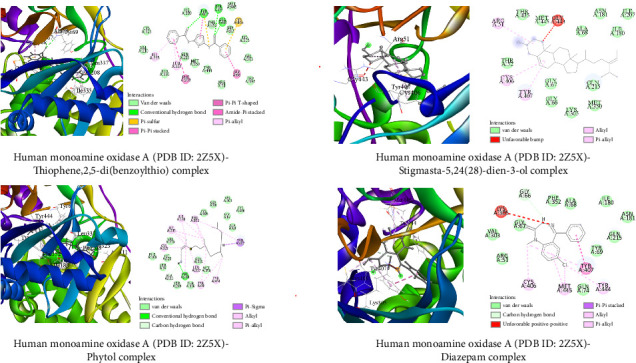
Top three metabolites and pefloxacin interactions with gyrase A (5ztj). The three primary metabolites and standard pharmaceuticals interact with the amino acids in the protein's active site through various sorts of bonds, as seen in the illustration.

The top three docked metabolites, along with the standard, are illustrated (both 3D and 2D) in Figure 9. The highest binding score showed by the thiophene,2,5-di(benzoylthio) with human monoamine oxidase A (PDB ID: 2Z5X) was −10.2 kcal/mol, while the standard diazepam was −8.8 kcal/mol. Stigmasta-5,24(28)-dien-3-ol and phytol showed binding energies of 8.8 kcal/mol and 8.7 kcal/mol, respectively.

##### 3.6.1.3. Molecular Docking Related to Antidiarrheal Activity

**Figure 10:**
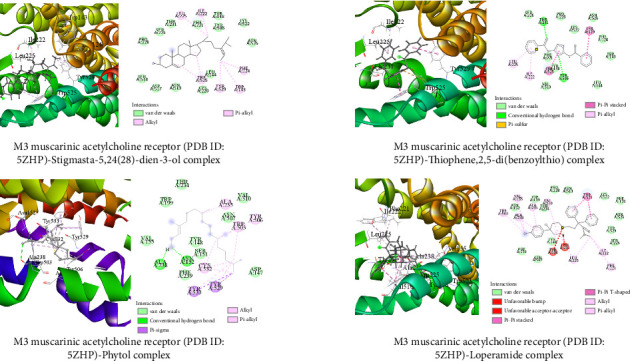
Molecular docking interactions of the top three metabolites and standard (loperamide) with M3 muscarinic acetylcholine receptor (PDB ID: 5ZHP). The three primary metabolites and standard drug interact with the amino acids in the protein's active site through various sorts of bonds, as seen in the figure.

The best-docked three metabolites are illustrated (both 3D and 2D) in Figure 10. Stigmasta-5,24(28)-dien-3-ol, thiophene,2,5-di(benzoylthio), and phytol showed binding activities of −8.8, −8.6, and −7.9 kcal/mol against M3 muscarinic acetylcholine receptor (PDB ID: 5ZHP), while the standard loperamide showed a binding score of −8.5 kcal/mol.

##### 3.6.1.4. Molecular Docking Related to Antibacterial Activity

**Figure 11:**
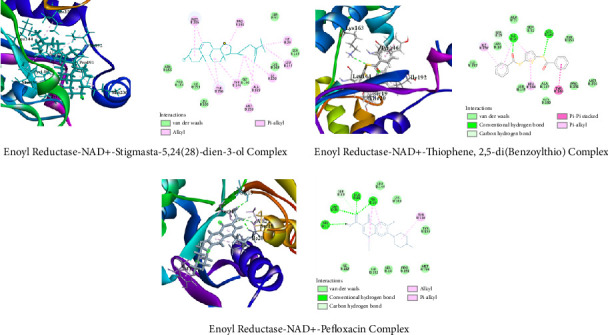
Top two metabolites and pefloxacin interactions with enoyl reductase NAD+ (1lx6). The two primary metabolites and standard pharmaceutical bind with the amino acids in the protein's active site through various sorts of bonds, as seen in the illustration.

**Figure 12:**
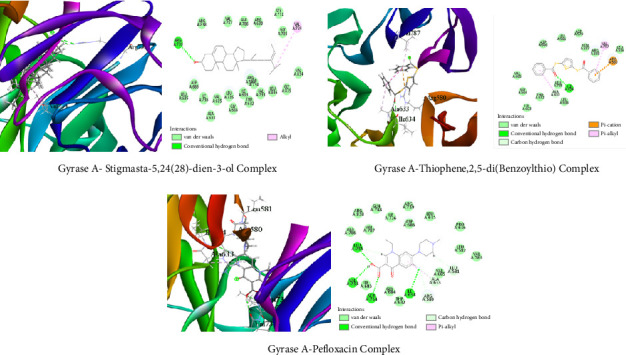
Top two metabolites and pefloxacin interactions with gyrase A (5ztj). The three principal metabolites and typical pharmaceuticals interact with the amino acids at the protein's active site through various sorts of linkages, as seen in the figure.

For *E. coli* enoyl reductase NAD+ (PDB ID: 1LX6), the top interacted compound was stigmasta-5,24(28)-dien-3-ol, which showed a binding energy of −7.9 kcal/mol, while the standard pefloxacin showed −7.2 kcal/mol. Another compound, thiophene,2,5-di(benzoylthio), demonstrated a binding energy of −7.4 kcal/mol against the protein (Figure 11). On the other hand, stigmasta-5,24(28)-dien-3-ol demonstrated the highest binding energy (8 kcal/mol) against gyrase A (PDB ID: 5ZTJ), which is greater than standard pefloxacin (−7 kcal/mol). Another compound, thiophene, 2,5-di(benzoylthio), has a binding energy of −6.1 kcal/mol against the protein (Figure 12).

## 4. Discussion

Seaweeds are notable for their numerous bioactive components and potential therapeutic benefits [34]. In our study, we looked into the therapeutic potential of the AECA for sedative, anxiolytic, antidiarrheal, and antimicrobial properties in rodent models.

The quantitative phytochemical analysis of the AECA was performed using GC-MS, where 12 metabolites were found between 2.5 and 32 min. The majority of the metabolites belong to fatty acids, followed by steroids, heterocyclic compounds, alcohols, esters, and amides. The AECA contains several chemicals with important pharmacological characteristics. According to Islam et al., phytol is a diterpene alcohol that produces sedative and anxiolytic effects by modulating GABAergic pathways [35]. N-Hexadecanoic acid (palmitic acid) exhibits broad-spectrum antimicrobial activity against both Gram-positive and Gram-negative bacteria, while its anti-inflammatory qualities may help prevent diarrhea by lowering intestinal hypermotility [36]. Tetradecanoic acid (myristic acid) has been shown by Kofi et al. to be antibacterial against infections like *S. aureus* [37]. Stigmasta-5,24(28) dien-3-ol, a phytosterol, has antibacterial action by rupturing microbial cell membranes [38]. Fumaric acid is also known to have antibacterial qualities against fungi and bacteria [38]. These findings highlight the diverse therapeutic potential of AECA.

The sedative potential of AECA was assessed using the OFT and HCT to investigate the effects of sedative and anxiolytic medications on behavior and locomotor activity [39]. OFT assesses exploratory behavior, which decreases when CNS depressants are administered. In HCT, decreased hole crossings reflect less locomotor activity and increased sedative properties [40]. A progressive significant decrease in the movement and hole crossing was seen in OFT and HCT after administration of an AECA 400 mg/kg dose, respectively. Similar CNS-depressant effects were shown with diazepam, which in both studies markedly decreased locomotor activity. *C. aerea* contains bioactive substances that have been demonstrated to affect the GABAergic system, specifically flavonoids and polyphenols, which are the reason for its sedative effect. This primarily arises through modulation of the GABAergic system. These bioactive compounds enhance inhibitory neurotransmission by binding to allosteric sites (e.g., benzodiazepine receptors) on GABA_A_ receptors, potentiating chloride ion influx and neuronal hyperpolarization, thereby reducing CNS excitability [41, 42]. Diazepam, which increases GABAergic neurotransmission and produces sleepy and anxiolytic effects, is one of the several sedatives that primarily target the GABA-A receptor [43]. The sedative qualities of marine algae, particularly species connected to the genus *Chaetomorpha*, have been demonstrated by supporting data from earlier research. As an illustration, *Chaetomorpha linum* has been shown to have a strong CNS depressant [44]. This is probably because the plant has a high concentration of bioactive metabolites like phenolic compounds, terpenoids, and alkaloids [45]. Studies focusing on the neuroprotective benefits of marine plants and their potential to cure anxiety and sleep problems, as well as traditional usage, lend credence to the sedative and hypnotic qualities of marine algae [45].

The EPM, HBT, and LDBT are three well-known behavioral tests that are commonly used to measure anxiety in mice, and were used to examine the anxiolytic properties of AECA [46]. Since rodents usually avoid open spaces because they are afraid of heights and exposure, an increase in time spent in and entries into the open arms of EPM suggests a decrease in anxiety [47]. The effects of AECA at 400 mg/kg were comparable to those of the typical anxiolytic diazepam, with a substantial increase in both the amount of time spent in the open arms and the number of entries. The dose-dependent decrease in time spent in and entry into the maze's closed arms corroborates this reduction in anxiety-like behavior and adds to the evidence of AECA's possible anxiolytic effects. HBT uses rodent exploratory behavior to measure anxiolytic effects. Head drooping and increased exploring are typically signs of decreased anxiety [46]. A dose-dependent anxiolytic effect was demonstrated by AECA. When compared to the control, the 400 mg/kg dose significantly increased the number of head dips, which was similar to the effect of diazepam. A two-chambered box with a light and dark side is used in the LDBT to detect anxiety-like behavior in mice. More time spent in the dark chamber is indicative of more anxiety in rodents, as they instinctively shun bright light. Longer stays in the bright areas are associated with reduced anxiety [48]. The number of transitions to the light compartment and the amount of time spent in the light compartment were both enhanced by the 400 mg/kg dose, and these results were very similar to the anxiolytic effects of diazepam. The bioactive components of AECA, including polyphenols, flavonoids, and alkaloids, which are known to alter GABAergic neurotransmission, are responsible for the anxiolytic effects that have been seen. The central nervous system's main inhibitory neurotransmitter, GABA, must be modulated to effectively manage anxiety. Similar to diazepam, these substances reduce anxiety-related behaviors by improving inhibitory neurotransmission through their interaction with GABA-A receptors. Additionally, they regulate monoaminergic pathways, e.g., by inhibiting monoamine oxidase (MAO) enzymes, which elevates GABA and serotonin levels while reducing catabolism of calming neurotransmitters. Complementary antioxidant activity further suppresses neuroinflammation and oxidative stress in sleep-regulating brain regions, stabilizing neural circuits involved in arousal [49, 50]. Other species, such as *C. linum*, have demonstrated comparable anxiolytic effects, demonstrating the medicinal use of marine algae [44].

The antidiarrheal activity of AECA was assessed using gastrointestinal motility and the castor oil–induced diarrhea test, two well-used methods for evaluating antidiarrheal effects and gut motility modulation [51]. In the test of castor oil–induced diarrhea, intestinal motility and fluid secretion are stimulated in mice, to cause diarrhea [51]. AECA 400 mg/kg demonstrated a strong antidiarrheal effect by significantly inhibiting diarrhea and reducing defecation, which were comparable to the effect of loperamide. The effect of AECA on the length of time a charcoal meal passes through the digestive tract is measured by the gastrointestinal motility test [51]. Both doses of AECA dramatically reduced gastrointestinal motility as seen by the peristaltic index, significantly dropping, which was comparable to loperamide. The bioactive components of AECA, like flavonoids and polyphenols, have protective effects on the gastrointestinal tract and are responsible for the antidiarrheal benefits that have been seen. Flavonoids and polyphenols exert antidiarrheal effects primarily through multitargeted actions on the gastrointestinal tract. They inhibit intestinal motility by blocking calcium channels and antagonizing muscarinic receptors (e.g., M3), which reduces smooth muscle spasms and hypermotility. Additionally, they inhibit enterotoxin secretion and bacterial adhesion through direct antimicrobial action against pathogens such as *E. coli* and *Vibrio cholerae*, thereby reducing infection-related fluid loss. Finally, they enhance mucosal barrier integrity by reducing inflammation through inhibition of NF-κB, COX-2, and pro-inflammatory cytokines (e.g., TNF-α, IL-1β), thereby decreasing vascular permeability and fluid secretion [52, 53]. Other marine algae have also been shown to have similar effects, highlighting *C. aerea's* possible therapeutic advantages [54].

The prevalence of antibiotic resistance, which reduces the number of available treatments and raises morbidity and mortality rates, makes bacterial infections a serious threat to public health. The growing occurrence of strains that are resistant to several drugs requires research into alternative antimicrobial agents, such as those that come from natural sources [55]. With inhibition zones of 14 mm and 13 mm, respectively, AECA (25 μg) demonstrated strong antimicrobial activity against Gram-negative bacteria in our investigation for *E. coli* and *S. typhi*, while the standard pefloxacin (5 μg) demonstrated an inhibition zone of 18 mm and 16 mm, respectively. The antimicrobial characteristics of *C. aerea* can be linked to its abundance of bioactive substances found in it, like polyphenols, flavonoids, and sulfated polysaccharides, which have inherent antibacterial characteristics [56] Several bioactive compounds found from the alga *C. aerea*, such as N-hexadecanoic acid, tetradecanoic acid, and stigmasta-5,24(28) dien-3-ol, demonstrated antimicrobial potentials, as shown by several studies [36, 37].

Molecular docking is a popular computational technique to forecast how ligands and proteins will interact, revealing information on binding affinities and interaction mechanisms [57]. In our study, metabolites found in the GC-MS analysis of AECA were docked against three proteins: the human GABAA receptor alpha1-beta2-gamma2 subtype (PDB ID: 6X3T), monoamine oxidase A (MAO-A, PDB ID: 2Z5X), and the M3 muscarinic acetylcholine receptor (PDB ID: 5ZHP) for sedative, anxiolytic, and antidiarrheal activities [58].

The GABAA receptor is crucial for inhibitory neurotransmission, which regulates sedation and anxiety. By binding to the neurotransmitter GABA, it activates chloride ion channels, causing neurons to hyperpolarize, resulting in reduced neuronal activity and calming effects [59]. Stigmasta-5,24(28)-dien-3-ol outperformed the typical sedative diazepam (−5.7 kcal/mol) in our docking investigation with a binding affinity of −7.0 kcal/mol. In addition, binding energies of −6.3 and −5.0 kcal/mol were shown for thiophene,2,5-di(benzoylthio) and phytol, respectively. These substances interacted with alkyl and pi-alkyl with VAL 227 and ILE 235, indicating that they may improve GABAergic transmission and provide sedative effects.

The oxidative deamination of neurotransmitters, including norepinephrine and serotonin, which affects mood and anxiety modulation, is catalyzed by the MAO-A enzyme [60]. Our findings showed that thiophene,2,5-di(benzoylthio) showed the highest binding affinity (−10.2 kcal/mol), whereas diazepam (−8.8 kcal/mol) suggests a strong interaction with MAO-A. Stigmasta-5,24(28)-dien-3-ol and phytol also demonstrated binding affinities of −8.8 and −8.2 kcal/mol, respectively. These substances established hydrogen bonds at the active site of the enzyme with ALA 68, TYR 69, and TYR 407, as well as other alkyl bonds, which may have prevented neurotransmitter deamination and enhanced the anxiolytic effects of AECA.

The gastrointestinal motility is regulated by the M3 muscarinic acetylcholine receptor for antidiarrheal action. When this receptor is inhibited, excessive motility is reduced, and the symptoms of diarrhea are lessened. Conversely, activation of this receptor might enhance intestinal contractions [61]. The maximum binding affinity (−8.9 kcal/mol) was demonstrated by stigmasta-5,24(28)-dien-3-ol, exceeding that of loperamide (−8.5 kcal/mol). Moreover, thiophene,2,5-di(benzoylthio) showed significant binding (−8.6 kcal/mol). These substances reacted with important amino acids such as TRP 525, LEU 225, and ILE 222 to produce hydrogen and alkyl bonds akin to those seen in loperamide, which inhibited the activity of the receptor and decreased intestinal motility.

*E. coli* Enoyl Reductase NAD+ (PDB ID: 1LX6), a crucial enzyme for the biosynthesis of fatty acids in *E. coli*, catalyzes the final step in the elongation of fatty acids [62]. Enoyl reductase is essential for conserving the integrity of the bacterial cell membrane; blockage of this enzyme can cause disruptions in membrane production, which can ultimately result in the death of the bacteria [63]. Considering the rising rates of antibiotic resistance, targeting enoyl reductase is a viable approach for creating novel antimicrobial drugs [64].

The highest binding affinity was demonstrated by stigmasta-5,24(28)-dien-3-ol (−7.9 kcal/mol), which was better than pefloxacin (7 kcal/mol). Gyrase A (PDB ID: 5ZTJ), topoisomerase II, is an important enzyme for DNA replication and maintenance in several bacteria, including *S. typhi* [65]. To validate *S. typhi's* susceptibility to AECA, this protein was studied in our in silico approach. To relieve torsional strain during DNA replication and transcription, it inserts negative supercoils into DNA. When gyrase A is inhibited, DNA replication is disrupted, which eventually results in bacterial cell death. Because of this, gyrase A is a crucial target for antibiotics, especially when treating infections brought on by *S. typhi*, the bacterium that causes typhoid fever [66]. The maximum binding energy (8 kcal/mol) of stigmasta-5,24(28)-dien-3-ol against gyrase A was found, surpassing that of conventional pefloxacin (−7 kcal/mol).

According to Ntie-Kang et al., virtual screening is an essential technique for assessing the theoretical ADME/T profiles of compounds before measuring their real activity against particular pharmacological targets. Every molecule that was chosen for our ADME analysis complied with Lipinski's Rule of Five, which is a crucial consideration when determining possible therapeutic targets [67]. Moreover, the ADME profiles of the docked compounds, analyzed using Lipinski's Rule of Five, revealed excellent pharmacokinetic characteristics, indicating their potential as therapeutic agents for sedation, anxiety, and diarrhea (Table 6). This in silico study highlights AECA's diverse neuropharmacological potential.

Results found in this scientific study indicate that the AECA can be a great source of sedative, anxiolytic, and antidiarrheal agents. Apart from the in vivo study, promising binding affinity with respective proteins was observed in the molecular docking study of the bioactive phytochemicals of the seaweed found in quantitative analysis. Drug-like properties of these metabolites were confirmed in ADMET analysis.

The primary limitations include the inability to isolate pure compounds from the crude extract, which impedes mechanistic understanding; a limited variety of tested microbial strains; and the preliminary status of in silico target interactions, necessitating validation through functional enzyme assays. Moreover, this sample size provides lower statistical power to detect modest effect sizes or interactions and increases vulnerability to outlier effects. Future research should focus on the isolation and confirmation of compounds through NMR or LC-MS/MS, comprehensive antimicrobial testing, and biochemical validation of the proposed activities of *C. aerea*. Also, confirmatory studies utilizing larger cohorts, determined by formal power analysis based on the effect sizes observed herein, are essential to validate the proposed potential of *C. aerea* extract robustly.

## 5. Conclusion

The findings of this scientific investigation suggest that the AECA may serve as an important mediator of sedative, anxiolytic, and antidiarrheal compounds. Apart from the in vivo study, promising binding affinity with the respective protein was observed in the molecular docking study of the bioactive phytochemicals of the seaweed found in quantitative analysis. Drug-like properties of these metabolites were confirmed in ADMET analysis. Biological studies of the *C. aerea* align with the computational approaches, which also support the results as well as make it a probable therapeutic candidate. Additional studies should be carried out to identify the bioactive metabolites, isolate the specific metabolites, evaluate their pharmacodynamics, and assess their activities on a large scale, with comprehensive antimicrobial testing and biochemical validation of these activities.

## Figures and Tables

**Table 1:** Total phenolic and flavonoid content in AECA.

Extract/standard	Total phenolic content (mg GAE/g extract)	Total flavonoid content (mg QUE/g extract)	IC_50_ value (μg/mL)
AECA	20.93 ± 1.05	15.78 ± 0.70	107.44
Ascorbic acid	—	—	72.62

**Table 2:** GC-MS identified metabolites with their characteristics.

Serial no.	Compound name	Molecular weight (g/mol)	Retention time (min)	Molecular formula	Concentration (%)
1	Thiophene,2,5-di(benzoylthio)	356.5	2.074	C_18_H_12_O_2_S_3_	0.97
2	Fumaric acid	116.07	7.762	C_4_H_4_O_4_	1.5
3	8-Heptadecene	238.5	9.054	C_17_H_34_	2.51
4	Hentriacontane	436.8	9.277	C_31_H_64_	1.07
5	Tetradecanoic acid, 10,13-dimethyl	228.37	11.612	C_14_H_28_O_2_	8.91
6	N-Hexadecanoic acid	256.42	12.047	C_16_H_32_O_2_	52.17
7	Phytol	296.5	13.388	C_20_H_40_O	16.93
8	3-Methyl-2(2-oxopropyl)furan	138.16	14.692	C_8_H_10_O_2_	0.83
9	9-Octadecenamide	281.5	15.561	C_18_H_35_NO	5.42
10	Bis(2-ethylhexyl) pthalate	390.6	16.915	C_24_H_38_O_4_	3.7
11	Cholesterol	386.7	21.212	C_27_H_46_O	0.79
12	Stigmasta-5,24(28)-dien-3-ol	412.7	22.64	C_29_H_48_O	0.88

**Table 3:** The effect of AECA on castor oil–induced diarrhea in mice (feces count).

Treatment (mg/kg)	Total number of feces	Inhibition of defecation (%)	Total number of diarrheal feces	Inhibition of diarrhea (%)
Negative control (0.1 mL/mouse)	14.33 ± 0.33	—	6 ± 0.57	—
Loperamide (5)	5.33 ± 0.33^∗∗∗^	62.80	2.66 ± 0.88^∗∗∗^	55.66
ACA 200	12.33 ± 1.85^∗^	13.95	4.33 ± 0.57^∗∗∗^	27.83
ACA 400	9 ± 1.52^∗∗^	37.19	3.66 ± 0.33^∗∗∗^	39

*Note:* Mean ± SEM (*n* = 5), with ^∗^*p* < 0.05, ^∗∗^*p* < 0.01, and ^∗∗∗^*p* < 0.001 indicating statistical significance.

**Table 4:** The effect of AECA on gastrointestinal motility in mice (charcoal marker).

Treatment (mg/kg)	Total length of intestine (cm)	Distance travel by charcoal (cm)	Peristalsis index (%)	Inhibition (%)
Control	50.33 ± 0.33	42.33 ± 1.20	84.10 ± 2.31	—
Loperamide (5)	52.33 ± 0.33	22.33 ± 0.33^∗∗∗^	42.66 ± 0.36^∗∗∗^	47.24
ACA 200	51.33 ± 0.33	32 ± 0.57^∗∗∗^	60.99 ± 0.48^∗∗∗^	12.6
ACA 400	50.66 ± 0.66	37 ± 0.57^∗∗∗^	54.55 ± 0.83^∗∗^	24.40

*Note:* Mean ± SEM (*n* = 5), with ^∗∗^*p* < 0.01, and ^∗∗∗^*p* < 0.001 indicating statistical significance.

**Table 5:** Molecular docking studies for GC-MS compounds.

PubChem ID	Compound name	Binding energy (kcal/mol)				
		6X3T	2Z5X	5ZHP	1LX6	5ZTJ
131750945	Stigmasta-5,24(28)-dien-3-ol	−7	−8.8	−8.8	−7.9	−8
5997	Cholesterol	−4.6	−7.3	−8.8	−7.4	−5
569794	Thiophene,2,5-di(benzoylthio)	−6.3	−10.2	−8.6	−7	−6
8343	Bis(2-ethylhexyl)pthalate	−4.8	−8.0	−7.4	−5.8	−5
5280435	Phytol	−5	−8.2	−7.9	−5.7	−5.9
12410	Hentriacontane	−4	−7.6	−7.1	−5.2	−5
11005	Tetradecanoic acid, 10,13-dimethyl	−4.1	−6.9	−6.7	−5.1	−4
985	N-Hexadecanoic acid	−4	−7.3	−6.7	−5	−4
5364555	8-Heptadecene	−3.5	−7	−6.5	−4.9	−4
5283387	9-Octadecenamide,(z)-	−3.6	−7.4	−6.3	−4.7	−5
545772	3-Methyl-2(2-oxopropyl)furan	−3.9	−5.8	−5.8	−4.7	−4
444972	Fumaric acid, 2-chlorophenyl	−3.6	−4.6	−5.3	−4.5	−4
3016	Diazepam	−5.7	−8.8	—	—	—
3955	Loperamide	—	—	−8.5	—	—
51081	Pefloxacin	—	—	−7.2	−7	—

*Note:* Human GABAA receptor alpha1-beta2-gamma2 subtype (PDB ID: 6X3T) for sedative, human monoamine oxidase A (PDB ID: 2Z5X) for anxiolytic, and M3 muscarinic acetylcholine receptor (PDB ID: 5ZHP) for antidiarrheal activities and antibacterial activity *E. coli* enoyl reductase NAD+ (PDB ID: 1LX6) and gyrase A (PDB ID: 5ZTJ).

**Table 6:** ADME/T properties of the GC-MS compounds.

Name of compounds	Absorption		Distribution		Metabolism	Excretion	Toxicity		Drug likeliness	Bioavailability
	Water solubility (log mol/L)	Intestinal absorption (% absorbed)	VDss (human) (log L/kg)	BBB permeability (log BB)	CYP3A4 substrate	Total clearance (log mL/min/kg)	AMES toxicity	Hepatotoxicity		
Thiophene,2,5-di (benzoylthio)	−6.22	92.677	0.01	0.059	Yes	−0.041	No	No	Yes	0.55
Fumaric acid	−0.642	71.771	−1.026	−0.127	No	0.89	No	No	Yes	0.85
8-Heptadecene	−8.277	91.208	0.644	0.948	Yes	1.929	No	No	Yes	0.55
Hentriacontane	−6.092	85.891	−0.016	1.222	Yes	2.188	No	No	Yes	0.55
Tetradecanoic acid, 10,13-dimethyl	−4.952	92.691	−0.578	−0.027	No	1.693	No	No	Yes	0.85
n-Hexadecanoic acid	−5.562	92.004	−0.543	−0.111	Yes	1.763	No	No	Yes	0.85
Phytol	−7.554	90.71	0.468	0.806	Yes	1.686	No	No	Yes	0.55
3-Methyl-2(2-oxopropyl)furan	−1.023	97.089	−0.06	0.055	No	0.668	No	No	Yes	0.55
9-Octadecenamide	−7.074	90.218	0.281	−0.389	Yes	1.959	No	No	Yes	0.55
Bis(2-ethylhexyl)pthalate	−6.47	92.45	0.36	−0.175	Yes	1.898	No	No	Yes	0.55
Cholesterol	−6.917	93.723	0.382	0.763	Yes	0.589	No	No	Yes	0.55
Stigmasta-5,24(28)-dien-3-ol	−6.715	94.642	0.179	0.764	Yes	0.619	No	No	Yes	0.55

## Data Availability

The data that support the findings of this study are available upon request from the corresponding author. The data are not publicly available due to privacy or ethical restrictions.

## Nomenclature

AECA: Acetone extract of *Chaetomorpha aerea*
OFT: Open field test
HCT: Hole cross test
EPM: Elevated plus maze
HBT: Hole board test
LDT: Light–dark box test
